# Optical characterization of laser-driven double Morse quantum wells

**DOI:** 10.1016/j.heliyon.2019.e02022

**Published:** 2019-07-10

**Authors:** E. Kasapoglu, S. Sakiroglu, H. Sari, I. Sökmen, C.A. Duque

**Affiliations:** a Faculty of Science, Department of Physics, Cumhuriyet University, 58140 Sivas, Turkey; b Dokuz Eylul University, Faculty of Science, Physics Department, 35390 Izmir, Turkey; c Faculty of Education, Department of Mathematical and Natural Science Education, Cumhuriyet University, 58140 Sivas, Turkey; d Grupo de Materia Condensada-UdeA, Instituto de Física, Facultad de Ciencias Exactas y Naturales, Universidad de Antioquia UdeA, Calle 70 No. 52-21, Medellín, Colombia

**Keywords:** Quantum mechanics, Optics, Non-resonant intense laser field, Optical absorption, Morse quantum well

## Abstract

In this study, the first-order linear, third-order nonlinear, and total absorption coefficients for the intersubband transition between the two lower-lying electronic levels in both symmetric and asymmetric double Morse quantum wells under the non-resonant high-frequency intense laser field are investigated. The study takes into account the effects of the structure parameters. The results show that the electronic and also accordingly optical properties of the structures which we focus on can be adjustable to obtain a convenient response to certain studies or purposes by changing the applied external field and optical intensity as well as structure parameters.

## 1 Introduction

Low dimensional structures such as quantum wells (QW), quantum wires, and quantum dots are very active research topics of nanoscience and nanotechnology that are the most important fields in the electronic and optical properties of solids and the design of high performance electronic, photonic, and optoelectronic systems. By choosing different potentials and using different methods, the bound states of these systems and also optical processes which is including both the linear and nonlinear properties based on transitions between the bound states have been intensively researched and are still being investigated. Due to the recent advances in material growth techniques, that enable possible to growth of semiconductor QWs with different confinement potentials, in addition to the well-known profiles such as rectangular, square, parabolic, semi-parabolic, and inverse parabolic single or double QWs with the shaped of symmetric and/or asymmetric under the external applied fields [1], [2], [3], [4], [5], [6], [7], respectively, the more realistic potentials such as Tietz-Hua, Morse, and Gaussian [8], [9], [10], [11] etc. have been produced, studied, and researches are still ongoing. External fields such as electric, magnetic, and intense laser field have become an interesting probe for studying the physical properties of the low-dimensional systems because of the external applied fields cause significant changes in the energy spectrum of the carriers. The main aim for the convenient choice of the QW profile is to manipulate the electronic structure so that the best reflect the atomic structure of the material and to provide the design of the new optoelectronic devices.

Double quantum well (DQW) systems have been studied intensively since they are promising systems both for fundamental researches and also industrial applications. As known, DQWs which characterized the bilayer systems are the semiconductor heterostructures exhibiting tunnel coupling. It means that the wave functions of an each well overlap in the barrier region and the subband energy levels become split off. This splitting depends on factors such as the well and barrier widths, and doping concentration. DQWs which consist of the various semiconductor materials appear frequently in the lasers emitting light in a wide range of wave length that is of great importance in optical communications and they are also taken into consideration as effective THz detectors [12], [13].

Additionally, potential energy functions for DQWs have been suggested to obtain information about diatomic molecules. The some of them are quartic [14], sextic [15], Razavy [16], Manning [17], and Morse [18] double well potentials which are called as quasi-exactly solvable (QES) potentials. These models provide a useful approximation for the potential energy of a diatomic molecule. Nonlinear optical properties of semiconductor QWs mainly depend on the asymmetry of the confinement potential and so the optical properties of the low dimensional semiconductor heterostructures with the Morse potential, due to the inherent asymmetric character, have been studied intensively [19], [20], [21], [22]. However, as far as we know, any study has not been reported on the optical properties of double Morse potential quantum wells in the literature. By considering this situation, we have investigated the optical properties both of the symmetric and asymmetric double Morse quantum wells (DMQW) under the non-resonant high-frequency intense laser field (ILF) by taking into account the effects of the structure parameters. It should be noted that the polarization of the incident radiation and the confining potential are oriented in the *z*-direction corresponding to the growth direction of the heterostructure. The method used to calculate the wave functions and energy levels of the system we have studied has been used in many studies before [23], [24], [25] and the degree of accuracy is quite sufficient. In order to test the convergence of the obtained results, also the Finite Elements Method (FEM) has been implemented to solve the eigenvalues differential equation.

The organization of the paper is as follows: Section 2 contains the presentation of the theoretical framework, in Section 3, we discuss the obtained results, and finally, in Section 4, we give our conclusions.

## 2 Theoretical framework

**Figure 1:**
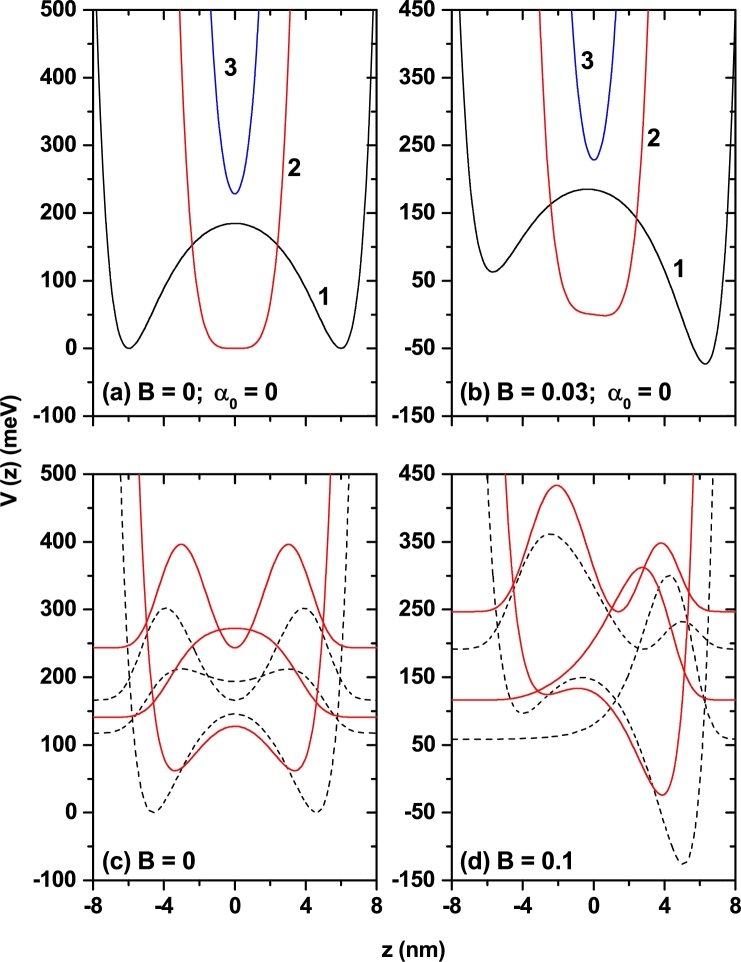
The pictorial views of the confinement potential for symmetric (a, c) and asymmetric (b, d) double Morse quantum well. In (a) and (b) three configurations of the *A*-parameter have been considered: 0 < *A* < 1 (1), *A* = 1 (2), and *A* > 1 (3). In (c) and (d), where *a* = 2 nm and *A* = 0.2, the results are for two values of the ILF-parameter: *α*_0_ = 0 (dotted lines) and *α*_0_ = 2 nm (solid lines). In (c) and (d) are depicted the curves for the density probability for the ground and first confined states.

In the effective mass approximation, the Hamiltonian for an electron confined into the region of a double Morse potential is given by

(1) $$H=\frac{p^{2}}{2\;m^{⁎}}+V_{DM}(z)\;,$$

where *p* is the momentum operator, $m^{⁎}$ is the electron effective mass, and $V_{DM}(z)$ is the double Morse confinement potential. The double Morse potentials, which are presented schematically in Figs. 1(a) and 1(b), are sum of two Morse potentials which is identical (different) for symmetric (asymmetric) potential oriented in back-to-back and centered at different points. The functional form of these potentials, for symmetric and asymmetric cases [26], is given as follows

(2) $$V_{SDM}^{ADM}(z)=V_{0}\;\left\{{\left[A\;\cosh\left(\frac{z}{a}\right)-1\right]}^{2}-B\;\sinh\left(\frac{z}{a}\right)\right\}\;,$$

where $V_{0}$ is depth of the quantum well, *a* is the parameter that changes the well and barrier widths, and *A* is a positive constant. When the *A*-parameter increases, the well and barrier widths become narrow while the central barrier height decreases. Thus, the DMQW has different shapes depending on the values of *A* as seen in Fig. 1(a). The three values A>1, A=1, and A<1 correspond to: *i*) the parabolic quantum well with the well bottom shifted towards higher values of the energy, *ii*) broad single quantum well which is very flat at the bottom, and *iii*) DMQW heterostructure, respectively. The constant *B* is the asymmetry parameter as seen from Fig. 1(b) and it is an arbitrary constant that can vary depending on the relevant material and purpose. Note that for negative (positive) values of the *B*-parameter, the effect on the confinement potential is very similar to the presence of an electric field parallel (antiparallel) oriented with respect to the growth direction of the heterostructure. In this paper, *a*, *B*, and *A* parameters will be called as the structure parameters.

In the presence of the ILF, symmetric and asymmetric double Morse potentials in Eqs. (2) turn into the form called laser-dressed potential

(3) $${\overset{˜}{V}}_{SDM}^{ADM}(z,\alpha_{0})=\frac{\overset{¯}{\omega }}{2\pi }\int_{0}^{2\pi /\overset{¯}{\omega }}V_{SDM}^{ADM}(z+\alpha_{0}\;\sin\overset{¯}{\omega }\;t)\;dt\;.$$

Using Eq. (2) into Eq. (3), the analytical expression for the laser-dressed potential gives

(4) $${\overset{˜}{V}}_{SDM}^{ADM}(z,\alpha_{0})=\frac{V_{0}}{2}\{2+A^{2}+A^{2}I_{0}\left(\frac{2\;\alpha_{0}}{a}\right)\;\cosh\left(\frac{2\;z}{a}\right)-2\;I_{0}\left(\frac{\alpha_{0}}{a}\right)\;\left[2\;A\;\cosh\left(\frac{z}{a}\right)+B\;\sinh\left(\frac{z}{a}\right)\right]\}\;,$$

where $I_{0}$ is the modified Bessel function of the first kind, $\alpha_{0}=(e\;A_{0})/(m^{⁎}\;\overset{¯}{\omega })$ is known as the laser-dressing or ILF parameter, $A_{0}$ and $\overset{¯}{\omega }$ are the magnitude of the vector potential and the angular frequency of the non-resonant ILF, respectively. The high frequency condition for the ILF is given as $\hbar \;\overset{¯}{\omega }>>E(\alpha_{0})$ which is the lowest order approximation and there is no other limitation on $\alpha_{0}$ apart from this condition, where $E(\alpha_{0})$ is the average excitation energy. The details of equations given above for dressed potentials and the nonperturbative approach based on the Kramers-Henneberger translation transformation developed to describe the atomic behavior in intense high-frequency laser field can be found in Refs. [27], [28], [29]. When the ILF is applied, the variation of the potentials for both symmetric and asymmetric DMQW are given in Figs. 1(c) and 1(d), respectively. As seen in these figures, with the effect of ILF the bottom of the quantum well shifts towards higher energy values, effective well width becomes narrow since the barrier width narrows, and this case also leads to an increase in electronic energies. Hereby, ILF will cause strong changes on the electronic and also optical properties of the structure.

The wavefunctions and the bound-state energies of the electron are obtained from the numerical solution of the time-independent Schrödinger equation corresponding to the DMQW, which has the laser-dressed confinement potential. Calculations have been made by using the diagonalization method [30]. Also, a FEM was used to obtain the eigenfunctions and eigenvalues of the problem. We have to stress that the agreement between the results obtained via the two methods was perfect. Then, the first-order linear, third-order nonlinear, and total absorption coefficients (ACs) for the transitions between the ground and first excited states of electron confined within DMQW by following the standard density matrix formalism combined with the perturbation expansion method are obtained, respectively, as follows [9], [19], [20], [21], [31], [32], [33], [34]

(5) $$\beta^{\left(1\right)}(E_{p})=\frac{E_{p}}{\hbar }\;\sqrt{\frac{\mu_{0}}{\varepsilon_{R}}}\;\frac{|M_{12}{|}^{2}\;\sigma_{v}\;\Omega_{12}}{{\left(E_{12}-E_{p}\right)}^{2}+\Omega_{12}^{2}}\;,$$

(6) $$\beta^{\left(3\right)}(E_{p},I)=-\;\frac{2\;I\;\beta^{\left(1\right)}(E_{p})}{\varepsilon_{0}\;n_{r}\;c}\;\frac{|M_{12}{|}^{2}}{{\left(E_{12}-E_{p}\right)}^{2}+{\Omega_{12}}^{2}}\times \left[1-\frac{|\Delta_{12}{|}^{2}}{|2\;M_{12}{|}^{2}}\;\frac{{\left(E_{12}-E_{p}\right)}^{2}-\Omega_{12}^{2}+2\;E_{12}\;(E_{12}-E_{p})}{{E_{12}}^{2}+\Omega_{12}^{2}}\right]\;,$$

and

(7) $$\beta (E_{p},I)=\beta^{\left(1\right)}(E_{p})+\beta^{\left(3\right)}(E_{p},I)\;,$$

where $\Delta_{12}=M_{22}-M_{11}$, $\varepsilon_{R}=n_{r}^{2}\;\varepsilon_{0}$ is the real part of the permittivity of the semiconductor material (with $\varepsilon_{0}$ being the vacuum permittivity and $\varepsilon_{r}=n_{r}^{2}$ being the dielectric constant of the semiconductor material), $\sigma_{V}$ is the carrier density in the system, $\mu_{0}$ is the vacuum permeability, $E_{12}=E_{2}-E_{1}$ is the energy difference between two electronic states, $M_{ij}=\langle \Psi_{i}|e\;z|\Psi_{f}\rangle$ (i,j=1,2) is the dipole matrix element between the eigenstates $\Psi_{i}$ and $\Psi_{j}$ for *z*-polarization of the incident radiation, $\Omega_{12}=\hbar /T_{12}$ is the relaxation rate (where $T_{12}$ is the relaxation time), *c* is the light speed in free space, and *I* is the optical intensity of the incident photon with the $E_{p}$ energy for the intersubband optical transitions.

We not that Eqs. (5) and (6) are not valid when using an incident photon whose energy is far from resonance for transitions between the ground state and the first excited state. In this work two types of incident radiation are considered: *i*) the first one with photon energy close to the resonance between the two lowest energy electronic states, and is the one that appears in Eqs. (5) and (6); *ii*) the second one is a high intensity and monochromatic radiation whose energy differs appreciably from the transition energy between the two lowest energy electronic states. It has been shown that the main effect of this radiation is to induce an effective change in the profile of the potential barriers (in their shape, dimensions, height, etc.) and it is through these changes that it induces modifications on the absorption coefficient. Changes in the potentials imply changes in the electronic states, both in the energies and in the wave functions.

## 3 Results and discussion

The parameter values in our numerical calculations are $\varepsilon_{r}=12.58$, $m^{⁎}=0.067\;m_{0}$ ($m_{0}=9.1\times 10^{-31}$ kg), $V_{0}=228$ meV, $n_{r}=3.2$, $T_{12}=0.4$ ps, $\mu_{0}=4\pi \times 10^{-7}$ Hm^−1^, $\sigma_{V}=3.0\times 10^{22}$ m^−3^, and $I=5.0\times 10^{8}$ W/m^2^. These parameters are suitable for GaAs/GaAlAs heterostructures.

**Figure 2:**
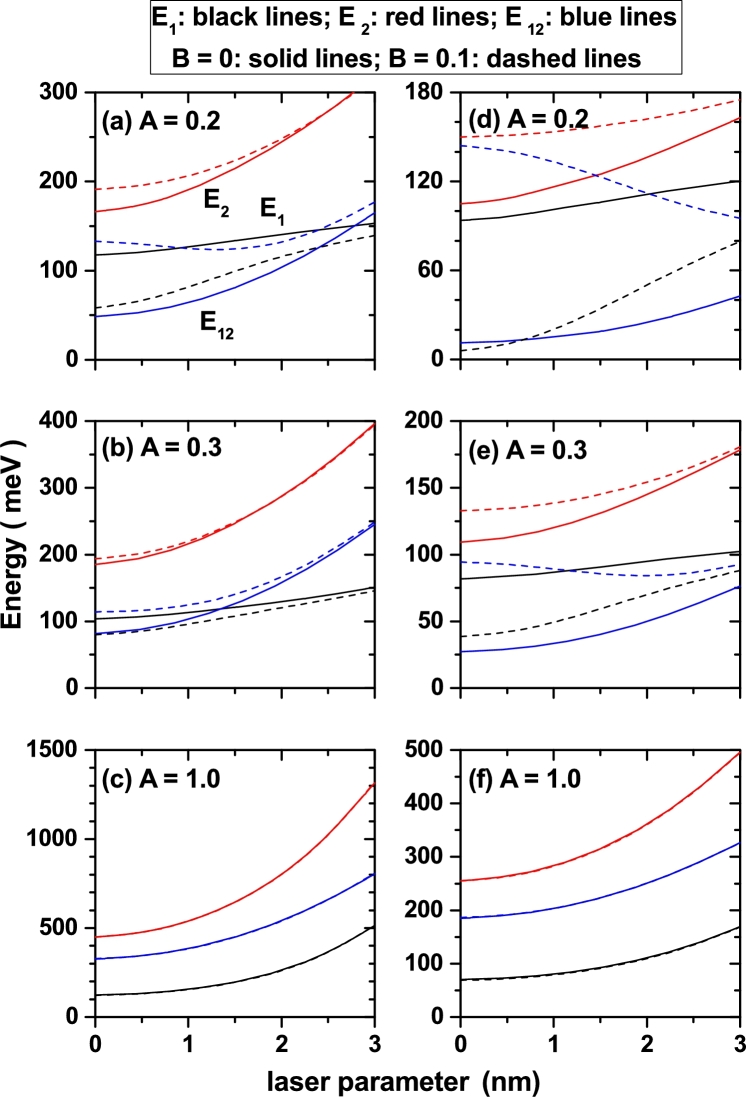
Energy of the ground (*E*_1_-black lines) and first excited (*E*_2_-red lines) energy levels and their difference (*E*_12_-blue lines) for a confined electron in the symmetric (*B* = 0, solid lines) and asymmetric (*B* = 0.1, dashed lines) DMQWs versus the *α*_0_-parameter with *a* = 2 nm (left-hand side column of panels) and *a* = 3 nm (right-hand side column of panels).

In Fig. 2 are presented the results for the energy of the ground and first excited energy levels, and their difference, for a confined electron in symmetric and asymmetric DMQWs versus the $\alpha_{0}$-parameter with two values of the *a*-structure parameter. Several values of the *A*-structure parameter have been considered. In all cases the energies and their differences are increasing functions of the ILF-parameter. Two exceptions to the rule, related with the energy difference, appear for dashed lines in panels (d) and (e). In these two cases the $E_{12}$ decreases with the laser parameter. Also in panel (a) the dashed line for the energy difference has a mixed behavior. The energies and their differences are increasing functions of the ILF-parameter because the presence of the ILF-parameter implies two combined effects: *i*) the decrease of the effective width of the structure and *ii*) the displacement towards higher values of the energy of the bottom of the potential well. The second effect becomes less sensitive as the *a*-structural parameter increases. It is clear that the second effect, the bottom shift of the potential well, can imply a rigid shift towards higher energies for both the fundamental and first excited states; implying that there is no change in the energy difference. In this case, the decrease of the well width with the ILF-parameter is responsible for the fact that in almost all the panels of Fig. 2 the energy difference is an increasing function with the ILF-parameter. Clearly, the decrease in well width has a greater effect on the excited state than on the fundamental state, since the wave function of the former extends over a larger region of space, which can feel a greater influence of the potential barriers that confine it. This explains why the growth rate with the ILF-parameter is higher for the energy of the excited state than for the fundamental state, resulting in the increasing value with the ILF-parameter of the $E_{12}$-difference. Keeping constant the $\alpha_{0}$, *a*, and *B* parameters, as *A* increases two main effects are observed: *i*) the reduction of the quantum well width and *ii*) the decrease of the central barrier, which is responsible for the appearance of the double well. In that sense, the *A*-parameter can be used to make the system evolve from isolated double wells (for small values of *A*), through coupled double wells (for intermediate values of *A*), and ending in single wells with high confinement of the carriers (for large values of *A*). Note that the effect of $\alpha_{0}$ and *A*-parameters is the same: reduction of the effective width of the structure and reduction of the central barrier.

The size of the QW is an increasing function of the *a*-parameter. Increasing the *a*-parameter, also increases the width of the well and decreases the confinement on the carriers, which explains why all the curves for $E_{1}$ and $E_{2}$, in the panels at the right-hand side column of Fig. 2, are displaced towards lower energy values; this with respect to the corresponding panel in the left-hand side column. Additionally, it is observed that when increasing the *a*-parameter (comparing panels in the right column with those in the left column), the excited state becomes less sensitive to the effects of the ILF-parameter, while the ground state retains its behavior. This fact explains why the effects on $E_{12}$ are reinforced by increasing the *a*-parameter. Another effect worth highlighting is that which arises from comparing the solid and dashed lines in each panel, i.e. when the asymmetry effects that are linked to the *B*-parameter. It is observed that when the *a*-parameter increases, that is to say, decreasing the confinement by the increase of the size of the structure, the effects of the asymmetry become more intense. As the *A*-parameter increases, i.e. the confinement increases due to the decrease in the spatial extension of the QW, the asymmetry becomes less relevant. Finally, when the *A*-parameter reaches its maximum value studied in this article (A=1), it is observed that the effects of asymmetry are null, which is consistent with the superposition of the solid and dashed curves in panels 2(c) and 2(f). The situation described here is perfectly in line with what is observed when electric fields are applied on carriers confined in QWs. In this sense, it can be stated that the effects of the electric field are not very relevant for structures in the range of a few tenths of the effective Bohr radius of the well materials.

**Figure 3:**
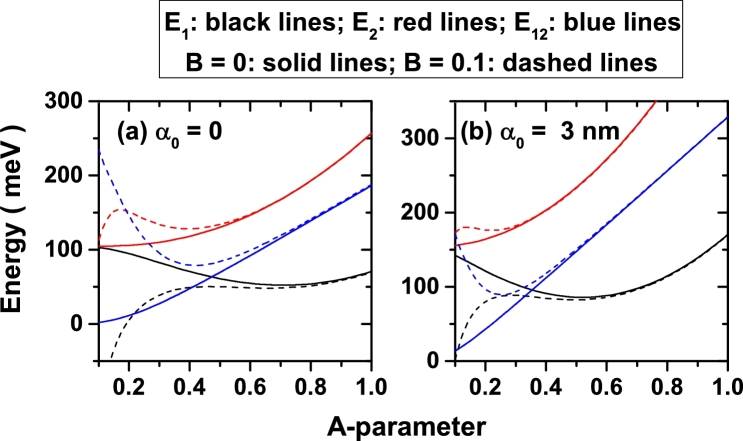
Energy of the ground (black lines) and first excited (red lines) energy levels and their difference (blue lines) for a confined electron in the symmetric (*B* = 0, solid lines) and asymmetric (*B* = 0.1, dashed lines) DMQWs versus the A-parameter with *a* = 3 nm and for two values of the laser parameter: *α*_0_ = 0 (a), and *α*_0_ = 3 nm (b).

In Fig. 3 we present the energies of the ground and the first excited state, as well as their difference, as a function of the *A*-parameter considering different configurations of the $\alpha_{0}$ and *B*-parameters keeping constant the value of the *a*-parameter, which is fixed in a=3 nm. In the first instance, the Fig. 3(a) shows the degeneration of the first two confined states for the symmetrical DMQW with A=0.1. This is due to the fact that the structure corresponds to two identical QWs, separated by a 20 nm distance, and isolated from each other by a central barrier of 200 meV. This degeneration is broken in three different ways: *i*) increasing the value of *A*-parameter, with which there is a reduction both in the distance between wells and in the height of the central barrier (in this case the wells are coupled and the degenerated levels are unfolded, as seen in the solid curves in Fig. 3(a)), *ii*) increasing the value of the ILF-parameter, which also decreases the distance between wells and the size of the central barrier (a situation that is clearly seen when comparing the solid curves in panels 3(a) and 3(b) when A=0.1), and *iii*) giving finite values to the *B*-parameter, which implies a break in the symmetry of the structure and therefore a split between states. In the case of symmetrical wells, note that the excited state is always an increasing function of the *A*-parameter, while the ground state one exhibits a minimum. This behavior has been observed for *p*-like states in quantum wells subjected to magnetic field effects. In asymmetric heterostructures, with B≠0, it is observed that, when varying the *A*-parameter, both the ground and the first excited state present a maximum, regardless of whether they are considered null or finite values of the laser parameter. These maxima are due to a few combined effect. First, by increasing the *A*-parameter, the width of the structure decreases and therefore the confinement increases, giving increasing values in the energy. There comes a time when the *A*-parameter implies a coupling between the two QWs, which increases the effective extension of the structure, decreasing the confinement and producing a fall in energy. From that point, again the increase of the *A*-parameter implies a reduction in the size of the well and the energies finally acquire the growing character. It is clear that those specific values of the *A*-parameter that imply changes in the behavior of $E_{1}$ and $E_{2}$, are specific for each state and for each value of the ILF-parameter. Finally, the Fig. 3 shows that the saturation effect induced by high values of the *A*-parameter depends on the magnitude of $\alpha_{0}$.

**Figure 4:**
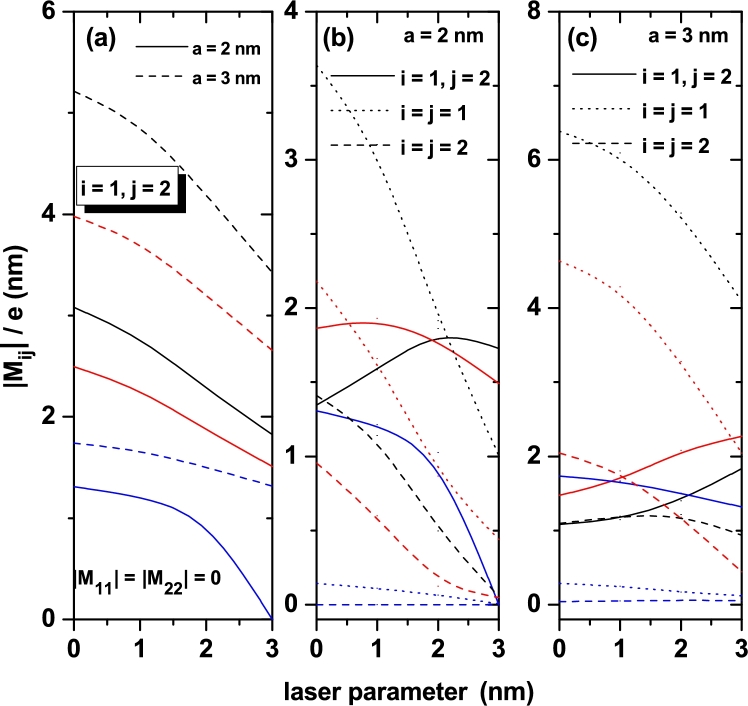
Diagonal and nondiagonal dipole matrix elements between the ground and first excited state of confined electrons in the symmetric (*B* = 0) (a) and asymmetric (*B* = 0.1) (b, c) DMQWs versus the laser-dressing parameters for three *A*-values: 0.2 (black lines), 0.3 (red lines), and 1.0 (blue lines).

According to Eqs. (5) and (6), the adequate description of the first and third order terms of the optical absorption, and therefore the total optical absorption value, depend on the exact knowledge of the diagonal and non diagonal terms of the electric dipole operator matrix element $M_{ij}$, which for linear polarization in the *z*-direction of incident radiation, which is responsible for stimulating the optical transitions, is proportional to the *z*-coordinate operator matrix element, which for discussion we will call $Z_{ij}=M_{ij}/e$. The non diagonal term, $Z_{12}=M_{12}/e$, is the determining factor in the first and third order correction in optical absorption, while the two diagonal terms $Z_{11}=M_{11}/e$ and $Z_{22}=M_{22}/e$ are only useful as a high-order correction factor to the nonlinear absorption term. In Fig. 4 are presented the results for the diagonal and nondiagonal dipole matrix elements between the ground and first excited state of confined electrons in the symmetric and asymmetric DMQWs as a function of the ILF- laser-dressing parameters for three values of the *A*-parameter.

In Fig. 4(a), where the structure corresponds to a symmetrical DMQW, and consistent with the even and odd character of the wave functions for the ground and first excited state, respectively, the diagonal terms $Z_{11}$ and $Z_{22}$ are identically zero. In Figs. 4(b) and 4(c), where asymmetric structures are considered, note the values close to zero for $Z_{11}$ and $Z_{22}$ when A=1. This is explained by the fact that for this value of the *A*-parameter, regardless of the asymmetry of the well, the system resembles a very thin parabolic QW (its width is less than 5 nm), as can be seen in curve 3 of Fig. 1(b). In this case, given the small extension of the well, the values of $Z_{11}$ and $Z_{22}$ are almost independent of the ILF parameter. In Figs. 4(b) and 4(c), the values of $Z_{11}$ and $Z_{22}$ are decreasing functions of the ILF-parameter since increasing this parameter reduces the width of the structure and induces a coupling between the two QWs that form the system, inducing an evolution towards a symmetrical structure. The latter due to the progressive reduction of the height of the central barrier with the increase of the ILF-parameter. Exactly the same effect is observed when comparing two curves of the same style on each of panels 4(b) and 4(c). Speaking now of the $Z_{12}$ terms in the three panels in Fig. 4, the following characteristics are observed. First, in Fig. 4(a), for all configurations, $Z_{12}$ are decreasing functions of the ILF-parameter. This is explained by the constant reduction of the effective width of the structure as $\alpha_{0}$ increases, which translates into a decrease in the spatial extent of the wave functions. Finally, this leads to a smaller spatial interval where can acts the *z*-factor which is involved in the calculation of the dipole matrix elements. This argument is also useful to explain why in all cases the value of $Z_{12}$ increases as the *A*-parameter grows. The presence of maximums in the curves of $Z_{12}$ in panels 4(b) and 4(c) is fundamentally associated with the coupling or decoupling effect between the two wells which can be associated with changes of the central barrier height, which is modified with the value of ILF-parameter.

**Figure 5:**
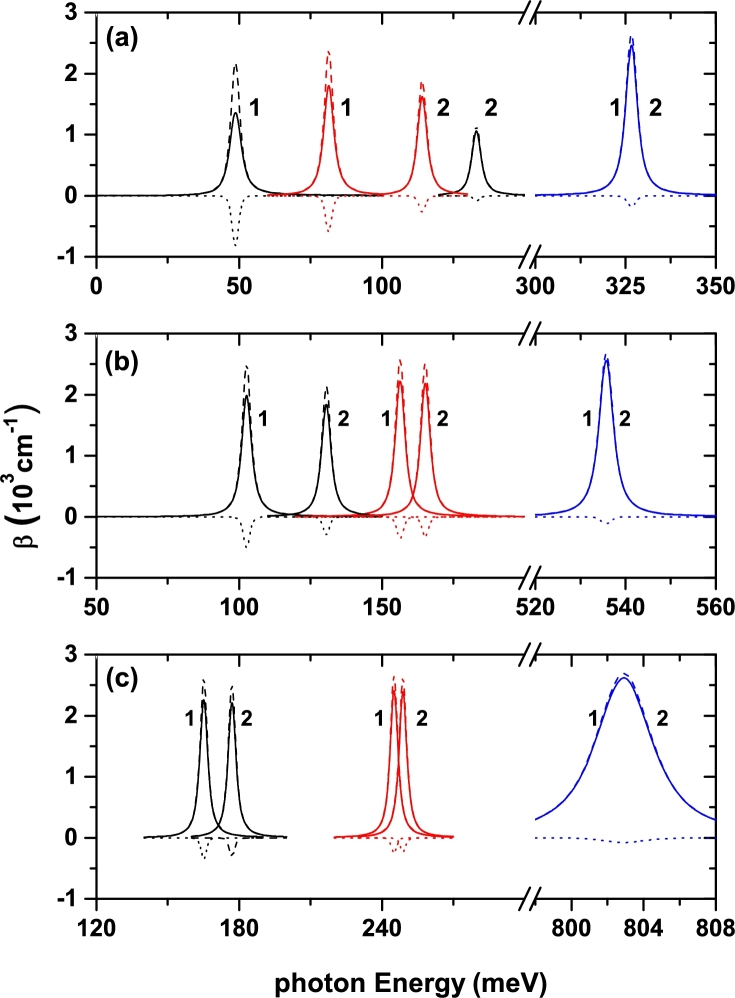
For *a* = 2 nm, the variations of linear (dashed lines), nonlinear (dotted lines), and total (solid lines) absorption coefficients of symmetric (*B* = 0, labeled with 1) and asymmetric (*B* = 0.1, labeled with 2) DMQWs versus the incident photon energy for different laser-dressing parameters: (a) *α*_0_ = 0, (b) *α*_0_ = 2 nm, and (c) *α*_0_ = 3 nm. Results are for *A* = 0.2 (black lines), *A* = 0.3 (red lines), and *A* = 1 (blue lines).

The variations of linear, third-order nonlinear, and total ACs in the symmetric and asymmetric DMQWs for a=2 nm versus the incident photon energy for different laser-dressing parameters $\alpha_{0}=0$, $\alpha_{0}=2$ nm, and $\alpha_{0}=3$ nm are given in Figs. 5(a-c), respectively. Results are for A=0.2 (black lines), A=0.3 (red lines), and A=1 (blue lines). For $\alpha_{0}=0$, when the *A*-parameter increases and in accordance with the results in Fig. 2, the peak positions of ACs for the symmetric (asymmetric) DMQW moves to higher (lower) photon energies with rising amplitudes due mainly to the increment in dipole matrix elements. For the value A=1, the absorption peaks overlap since the structure transforms into a single quantum well, but there is a very small difference between the magnitude and position of two peaks due to the *B*-asymmetry parameter. In Fig. 5(b), the peak positions shift towards higher photon energies with increasing amplitudes as the *A* increases due to the effect of the ILF-parameter. In Fig. 5(c), for also the other *A*-values taken into account as in the case of A=1, the absorption peaks of both the symmetric and asymmetric DMQWs begin to overlap, and also the values of the energy difference between the first two energy levels in both DMQWs begin to be the same, this behavior occurs in smaller ILF values with increasing *A* values.

**Figure 6:**
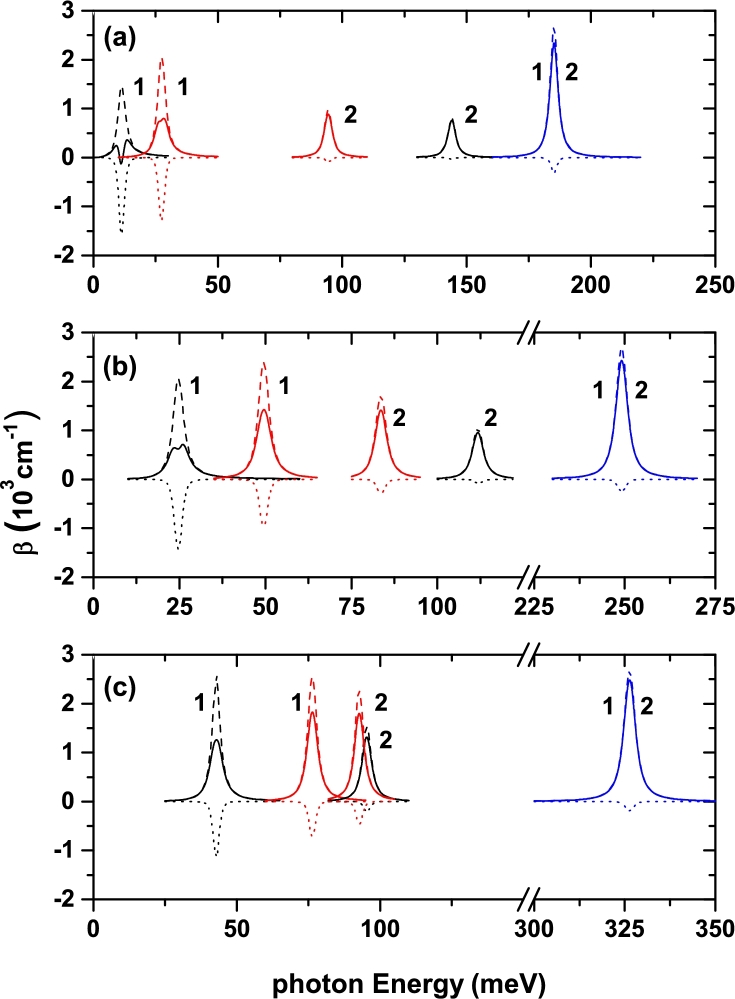
For *a* = 3 nm, the variations of linear (dashed lines), nonlinear (dotted lines) and total (solid lines) absorption coefficients of symmetric (*B* = 0, labeled with 1) and asymmetric (*B* = 0.1, labeled with 2) DMQWs versus the incident photon energy for different laser-dressing parameters: (a) *α*_0_ = 0, (b) *α*_0_ = 2 nm, and (c) *α*_0_ = 3 nm. Results are for *A* = 0.2 (black lines), *A* = 0.3 (red lines), and *A* = 1 (blue lines).

Figs. 6(a-c) are the same as in Figs. 5(a-c), but the results are for a=3 nm. As the *a*-parameter increases, the absorption peaks move to the lower photon energies with decreasing magnitudes for all *A* values taken into account, since the well and barrier widths increase and thus, energy difference between related energy levels decreases. Also for the other *A*-values, except for A=1, bleaching effect is observed on total ACs for the symmetric DMQW, but not for the asymmetric DMQW. This is due to the negative contribution of third-order nonlinear term. In the case of symmetric DMQW, the resonant peaks split up into two separate peaks (bleaching effect), since difference between the magnitudes of linear and third-order nonlinear ACs is small. As ILF strength increases, firstly for A=0.2, then for A=0.3 the bleaching effect begins to disappear. Furthermore, all AC peak positions shift towards blue and their magnitudes rise with the ILF-parameter. The reason for shift towards blue or red in AC peaks, is strictly associated with the $E_{12}$-energy differences between the first two subband energies in the symmetric and asymmetric DMQWs as a function of the $\alpha_{0}$, *a*, *A*, and *B* parameters, results that were previously presented and discussed in Figs. 2 and 3. As mentioned above, in the case of A=1 the structure turns into a single quantum well, therefore $E_{12}$ values become the same for both symmetric and asymmetric structures, thus the absorption peak positions corresponding to both structure for each an *a*-value become overlapped. Furthermore, the increment in $E_{12}$ for a=2 nm is greater and sharper more than that of a=3 nm under the ILF and consequently the blue-shifts are magnified.

The peak positions of ACs are located in larger photon energies for the a=2 and A=1 values of the structure parameters. For A=0.2, the energy difference corresponding to the symmetric DMQW (asymmetric DMQW) for the two *a*-values increases (decreases) with increasing laser-dressing parameter, then the energy difference between the related energy levels in the asymmetric DMQW starts to increases for a=2 nm after a certain $\alpha_{0}$-value, while it always decreases for a=3 nm. Since the energy difference between the first two subband states in asymmetric DMQW is greater than that of symmetric DMQW, the peak positions of the ACs relating to asymmetric DMQW place in high photon energies as seen in Figs. 5(a) and 6(a). For A=0.3, $E_{12}$ decreases for only a=3 nm in the asymmetric DMQW with the laser-dressing parameter while it increases for the other cases. Furthermore, as $\alpha_{0}$ increases $E_{12}$ values approach to each other for both DMQWs, this behavior is more pronounced for a=2 nm. Thus, absorption peak positions of both DMQWs approach to each other as seen in Figs. 4 and 5. It should be noted that the bleaching effect is observed in the case of $E_{12}<25$ meV as seen in Figs. 6(a) and 6(b). This is an expected result, because the bleaching effect is observed in the absorption spectrum if the energy difference between any two states is sufficiently small and optical intensity which leads to the intersubband optical transitions is sufficiently high. It is already known that the bleaching effect disappears by using a smaller optic intensity than the one used here when the energy difference between the two energy levels is small.

## 4 Conclusions

In the present study, we have investigated the absorption coefficients for the intersubband transition between two lower-lying electronic levels (1-2) of the symmetric and asymmetric double Morse quantum wells under the non-resonant high-frequency intense laser field by taking into account the effects of the structure parameters and intense laser field. Eigenvalues and eigenfunctions of the electron confined within the structure under the intense laser field are obtained from the solution of the time independent Schrödinger equation by using the effective mass and parabolic band approximations. The optical response is treated with the use of a two-level approach in the density matrix expansion. Our results show that the optical properties of the symmetric and asymmetric double Morse quantum wells are extremely responsive to ILF and to the structure parameters, in particular, to the asymmetry parameter.

## Declarations

### Author contribution statement

E. Kasapoglu: Conceived and designed the experiments; Wrote the paper.

S. Sakiroglu: Performed the experiments.

H. Sari, I. Sokmen: Analyzed and interpreted the data.

C.A. Duque: Analyzed and interpreted the data; Wrote the paper.

### Funding statement

This work was supported by Colombian Agencies: CODI-Universidad de Antioquia (Estrategia de Sostenibilidad de la Universidad de Antioquia and projects “Propiedades magneto-ópticas y óptica no lineal en superredes de Grafeno” and “Estudio de propiedades ópticas en sistemas semiconductores de dimensiones nanoscópicas”), and Facultad de Ciencias Exactas y Naturales-Universidad de Antioquia (CAD exclusive dedication project 2018-2019). The authors also acknowledge the financial support from the Colombian Agency *El Patrimonio Autónomo Fondo Nacional de Financiamiento para la Ciencia, la Tecnología y la Innovación, Francisco José de Caldas–**COLCIENCIAS* (project 80740-173-2019).

### Competing interest statement

The authors declare no conflict of interest.

### Additional information

No additional information is available for this paper.
